# Three-dimensional X-ray micro-velocimetry

**DOI:** 10.1107/S0909049510040434

**Published:** 2010-11-23

**Authors:** Wah-Keat Lee, Kamel Fezzaa, Tomomasa Uemura

**Affiliations:** a Advanced Photon Source, Argonne National Laboratory, Argonne, IL 60439, USA; b Kansai University, 3-3 Yamate, Suita, Osaka 564-8680, Japan

**Keywords:** X-ray imaging, 3D velocimetry, stereo imaging, 3D particle tracking, 3D flow velocimetry

## Abstract

A direct measurement of three-dimensional X-ray velocimetry with micrometer spatial resolution is presented. The key to this development is the use of a Laue crystal as an X-ray beam splitter and mirror. Three-dimensional flow velocities in a 0.4 mm-diameter tubing were recorded, with <5 µm spatial resolution and speeds of 0.7 mm s^−1^. This development paves the way for three-dimensional velocimetry in many cases where visible-light techniques are not effective, such as multiphase flow or flow of optically opaque liquids.

## Abstract 2

Three-dimensional X-ray velocimetry with micrometer-level resolution is demonstrated.

## 1. Introduction

Particle image velocimetry (PIV) and particle tracking velocimetry (PTV) using visible light are well established techniques for measuring flow fields (Adrian, 1991 ▶, 2005 ▶). In a typical set-up, a thin sheet of light illuminates a cross section of the sample and two-dimensional images of the illuminated plane are recorded. In cases where there is sufficient symmetry and where simple assumptions (for example, Poiseulle flow) on flow properties can be made, it is possible to deduce a three-dimensional flow map from the two-dimensional data (Alanentalo *et al.*, 2008 ▶). For steady-state systems, this can be accomplished by scanning the focal plane through the fluid. In general, however, multiple two-dimensional views are required to yield three-dimensional flow information (Bown *et al.*, 2006 ▶; Maas *et al.*, 1993 ▶).

Visible-light velocimetry techniques, however, have several limitations. Opaque systems such as *in vivo* blood flow are difficult or impossible to measure. Multiple scattering, refraction and reflection are major problems; for example, highly turbulent bubbly flow cannot be investigated because the fluid is effectively opaque owing to the scattering from the liquid–bubble interfaces. Visible-light techniques measure the flow in a cross-sectional plane of the sample; the thickness of the sampled plane depends on the depth of field of the lens and the illumination. For large magnification lenses, the corresponding depth of field becomes very small, and the tracer particles may not remain within the depth of field. This is a particularly difficult problem for non-laminar flows. Finally, for micrometer-resolution velocimetry, the tracer particles have to be correspondingly small and become comparable (within an order of magnitude) with the wavelength of the visible light. In this Rayleigh regime, the scattered intensity is highly dependent on particle size (Adrian, 1991 ▶; Dyuzheva *et al.*, 2002 ▶; Khlebtsov, 2003 ▶). This can pose a challenge to the dynamic range of the detectors and in the subsequent analysis.

Many problems associated with the use of visible light can be solved by the use of X-rays. X-ray interaction with matter is weak and multiple scattering is negligible. X-rays easily penetrate optically opaque systems. Refraction and reflection angles are very small (∼10^−6^ rad) and so X-rays essentially travel in straight lines. Multiphase systems can be investigated. Since the X-rays penetrate the entire sample and the image is a two-dimensional projection of the sample in a plane perpendicular to the direction of the X-rays, the image is not confined to the depth of field of the lens as in the visible-light systems. Finally, since the X-ray wavelengths (∼10^−10^ m) are much smaller than the typical size of tracer particles (∼10^−6^ m for micrometer-level spatial resolutions), there are no differences in scattering owing to particle sizes.

Until recently, however, X-ray velocimetry measurements have been limited by the relatively low X-ray flux of laboratory-based X-ray sources. Only low spatial (0.5–1.0 mm) resolution images can be recorded (Seeger *et al.*, 2001 ▶; Lanzillotto *et al.*, 1996 ▶; Lee *et al.*, 2004 ▶). Another challenge of X-ray systems is the availability of appropriate tracer particles. Conventional X-ray image contrast depends on the differences in absorption. Thus, for good contrast, highly absorbing metallic particles are ideal. However, these particles typically have significantly higher densities than the fluid and thus there is the problem of buoyancy. As a result, the image quality from these laboratory-based X-ray systems is generally inferior to those from visible-light systems and the range of applications is limited. With the advent of synchrotrons where the X-ray flux is orders of magnitude higher than laboratory-based sources, micrometer-level spatial resolution imaging became possible and its application to velocimetry has been demonstrated (Lanzillotto *et al.*, 1996 ▶; Lee & Kim, 2003 ▶, 2005 ▶). Recently, with third-generation synchrotrons, two-dimensional X-ray velocimetry *without* trace particles has been demonstrated in a dense liquid spray by tracking the phase-contrast features with micrometer-level spatial resolution (Kim & Lee, 2006 ▶; Irvine *et al.*, 2008 ▶) *and* submicrosecond temporal resolution (Wang *et al.*, 2008 ▶).

Synchrotron-based X-ray micrometer-resolution velocimetry measurements, however, have thus far been mostly limited to two dimensions. X-ray imaging is a projection technique and, unlike visible-light techniques, there is no depth resolution. As in visible-light techniques, for the cases of high symmetry and where assumptions on flow properties are made, it is possible to deduce three-dimensional flow information from the two-dimensional X-ray projected images (Im *et al.*, 2007 ▶; Irvine *et al.*, 2010 ▶; Fouras *et al.*, 2007 ▶). Thus far, the only three-dimensional X-ray direct micro-velocimetry measurement that does not require an *a priori* knowledge of the flow or an assumption of axial symmetry has been made by combining velocimetry with tomography (Dubsky *et al.*, 2010 ▶). Two-dimensional projection images are recorded for different sample rotation angles relative to the incident X-ray beam, and the three-dimensional flow field is reconstructed tomographically. The dis­advantage of this technique is that the flow fields must be constant over the time it takes for a complete tomographic data set; in the case of Dubsky *et al.* (2010 ▶), this time is ∼10 s. This limits the technique’s applicability. As far as we know, there has not been an X-ray imaging technique for velocimetry that directly *measures* the three-dimensional velocity field with micrometer-level spatial resolution that is generally applicable (even in cases of unsteady/unpredictable flow), without the need for symmetry and/or *a priori* knowledge of the flow.

**Figure 1:**
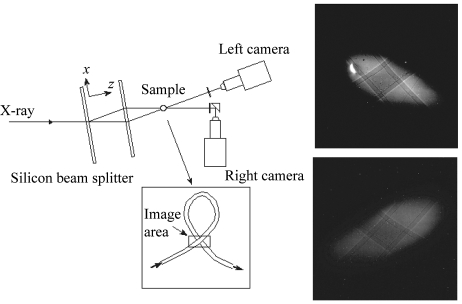
Schematic and coordinate system of the set-up. The loop was oriented to be roughly in the *xy* plane. The bright spot on the left side of the top X-ray image is due to a blemish on the X-ray scintillator.

In this paper we present a direct three-dimensional X-ray velocimetry measurement technique with micrometer-level spatial resolution at a synchrotron. The key to this development is the use of a double-Laue crystal to generate two simultaneous incident beams onto the sample with approximately 20° angle between them. In this first demonstration of the technique we achieved better than 2 µm lateral and 5 µm longitudinal resolution in particle position with an image acquisition rate of 60 Hz. The speed measurement errors are approximately 0.02 mm s^−1^ and 0.04 mm s^−1^ in the lateral (*xy* plane) and longitudinal (*z*) directions (see coordinate system in Fig. 1 ▶), respectively.

## 2. Experiment and results

The experiment was carried out at the XOR-32-ID beamline at the Advanced Photon Source. A vertically deflecting double-crystal Si (111) Bragg monochromator delivered 18 keV X-rays into the experimental station with ∼10^13^ photons s^−1^ mm^−2^ incident flux. In the station, the first blade of a horizontally scattering Si (220) double-Laue crystal splits the beam into the transmitted and reflected directions (20.6° separation). The second Laue blade deflects these beams so that they intersect at the sample position. The Laue blades are ∼0.45 mm thick. The crystal was cut from an ingot of single-crystal float-zone silicon (Topsil, USA). The combination of the vertically scattering monochromator and the horizontally scattering Laue crystal results in a tilted ellipsoid-shaped beam (Fig. 1 ▶). A cerium-doped yttrium aluminium garnet scintillator converted the X-rays into visible light that is then imaged onto the detectors *via* 5× Mitutoyo microscope objective lenses. Two synchronized high-speed cameras (Photron APX-RS, 17 µm pixel size, 1 K × 1 K pixels) were used to record the images. The demagnified pixel sizes were ∼3.87 µm. Images were recorded at 60 Hz.

Calibration of the camera orientation angle and magnification was performed using a 400-mesh gold transmission electron microscope (TEM) grid. The parallax angle (relative to the grid normal) for each camera is the inverse cosine of the ratio of the measured horizontal period (in pixels) to the measured vertical period of the TEM grid. The sum of the two parallax angles is the stereo angle, θ. This method of calculating the stereo angle differs by ∼6% from the known X-ray energy and silicon 220 lattice spacing, and, compared with other sources of error (see below), is inconsequential. Calibration of the sample *z*-position was performed using a sample made by wrapping a thin wire around a pencil lead of known diameter.

For this demonstration study we used an open-loop flow system consisting of a 0.4 mm-inner-diameter silicone rubber tubing (Fig. 1 ▶) attached at one end to a KDS 1000 syringe injector. The fluid used was 300 cSt silicone oil, and 10–30 µm particles (aluminium or solder) were used as tracers. By appropriately routing the rubber tubing, different flow geometries suitable for three-dimensional PTV demonstration can be easily achieved. Here, we limit our discussion to a simple cross (X) type of flow (see Fig. 1 ▶) with solder tracer particles. The plane of the cross was oriented such that it was roughly parallel to the planes of the Laue crystals.

Raw images were first corrected for relative camera displacement, parallax and roll, based on results of the calibration process described above. The two-dimensional coordinates of the trace particles were identified in each corrected projection and from them the coordinates of the particles were calculated. Owing to insufficient contrast, manual identification of the trace particles was necessary in this demonstration study. The locations of the inner and outer diameters of the tube walls were similarly measured. The velocity fields were measured by tracking the particles’ positions ten frames (166.67 ms) later.

The resolution of the technique depends on how accurately the positions of the tracer particles can be measured. The particles are located manually, background, magnification and distortion corrected, and binarized. The centroid of the binarized particle image was used as the particle position. Although the particle sizes used in the fluid ranged from 10 to 30 µm in diameter, the average size of the particles that were actually tracked were towards the smaller end of the distribution. This is because the larger and heavier particles did not make it through to the section of the flow system that was imaged. On average, the particle size that was tracked covered an area of about ten pixels (∼14 µm diameter). The error in the centroid position in the image can be estimated as the inverse of the square root of the area, which is about 0.3 pixels. In the sample coordinate system (Fig. 1 ▶) the particle coordinates are given by
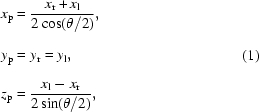
where the subscripts l and r denote the left and right camera coordinates, respectively, and θ is the stereo angle (20.6°). Thus, the error in the particle position in the *xy* plane is ∼0.3 pixels (1.2 µm) while that for the *z*-direction is ∼0.9 pixels (3.5 µm). The particle displacement is given by
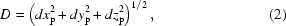
where *dx*_p_, *dy*_p_, *dz*_p_ are the displacements in the particle coordinates between the frames. For our analysis we used a ten-frame interval (166.67 ms). The error in the displacement is thus given by
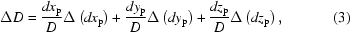
where, from above, 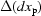 = 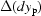 = 2 × 0.3 pixels = 0.6 pixels, and 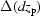 = 2 × 0.9 pixels = 1.8 pixels. The error on the speed depends on the component-vectors and speed. If the velocity vector is purely in the *xy* plane, the worst-case scenario is where the flow is along the *y* = *x* direction (which is approximately our set-up), 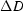 = 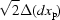, which results in an error in the speed of ∼0.02 mm s^−1^. If the velocity vector is purely in the *z* direction, 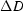 = 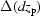, which results in an error in the speed of ∼0.04 mm s^−1^.

**Figure 2:**
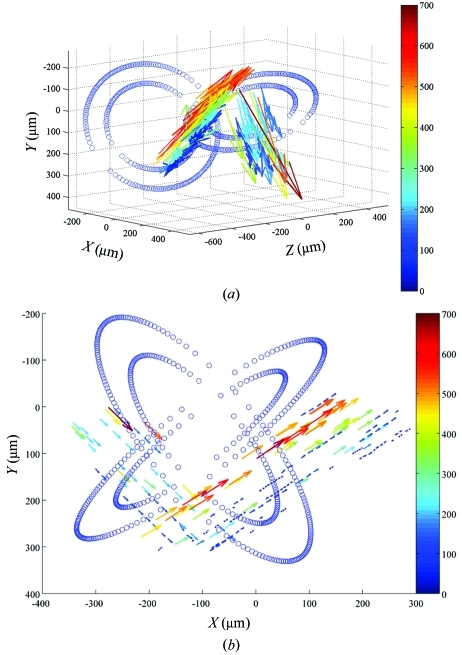
(*a*) Three-dimensional rendition of measured velocity fields. Inner and outer diameters of the two crossed tubes are shown (open circles). Arrows represent the flow vectors; length and color of the vector denotes the speed. The color bar shows the speed in µm s^−1^. (*b*) Projected velocity on the *xy* plane. The arrows represent the velocity vectors in the *xy* plane; length and color of the vectors represent the in-plane speed. Color bar speed units are µm s^−1^. Open circles denote the inner and outer diameters of the tubes.

Fig. 2 ▶ shows the results from a cross-flow geometry. The tube walls and the positions and velocities of the tracked particles are shown in Figs. 2(*a*) (three-dimensional display of flow) and 2(*b*) ▶ (flow projected onto the *xy* plane). Owing to the density of the tracer particles (solder), the tracked particles were heavily biased towards the lower edge of the tubes. As a result, the measured tracer-particle speeds here are probably not a faithful representation of the actual fluid speeds. This will be rectified in the future by using X-ray compatible neutrally buoyant tracers such as metal-coated hollow glass spheres. Alternatively, it would be interesting to combine this stereo technique with X-ray PIV measurements that do not require tracer particles (Kim & Lee, 2006 ▶; Irvine *et al.*, 2008 ▶). This would be advantageous in that the question of whether the tracer particles truly reflect the underlying fluid flow would not arise.

## 3. Conclusion

In summary, we have demonstrated a direct three-dimensional X-ray velocimetry measurement with micrometer-level spatial resolution. This is a significant improvement over current X-ray velocimetry capabilities in that it does not require symmetry in the flow, or *a priori* knowledge of the flow. In addition, unlike tomographic techniques, it can be applied to non-equilibrium flow conditions. Possible applications include three-dimensional micro-flow patterns in systems where visible-light techniques are not effective such as ferrofluid flow, microchannel flow where reflection/refraction from microchannel walls makes it difficult to measure the near-wall flow properties. Future improvements include plans to use tracer particles that have better contrast that would allow for software-based particle tracking, and are neutrally buoyant, and an upgrade of the X-ray optics to improve the transmission efficiency. Adapting this technique to speckle-tracking velocimetry would also be a promising development.
